# A Comprehensive Review of Zero-Dimensional Carbon-Based Nanomaterials in Anti-Corrosive Coating Applications: A Combined Quantitative and Qualitative Analysis

**DOI:** 10.3390/molecules31091521

**Published:** 2026-05-03

**Authors:** Xiaochuan Liu, Gaofei Kong, Shengbin Li, Bo Zhou, Chuang He, Haijie He, Shuang E

**Affiliations:** 1 School of Architectural Engineering, Yan’an University, Yan’an 716000, China; xiaochuanliu@yau.edu.cn (X.L.); hhh1233210323@163.com (S.L.); 2 Yan’an Medical College, Yan’an University, Yan’an 716000, China; konggaofei@yau.edu.cn; 3 Department of Civil, Construction, and Environmental Engineering, Iowa State University, Ames, IA 50011, USA; bozhou@iastate.edu; 4 School of Civil Engineering and Architecture, Taizhou University, Taizhou 318000, China; 5 College of Civil and Architectural Engineering, Zhejiang University, Hangzhou 310058, China; 6 College of Optical Science and Engineering, Zhejiang University, Hangzhou 310027, China; 7 College of Life Science, Dalian Minzu University, Dalian 116600, China

**Keywords:** zero-dimensional carbon-based nanomaterials, anti-corrosive coatings, nanodiamond-based coatings, fullerene-based coatings, carbon dot-based coatings

## Abstract

Anti-corrosive coatings are among the most widely used methods for corrosion protection. Zero-dimensional (0D) carbon nanomaterials have attracted increasing attention due to their advantages, such as small size, high specific surface area, ease of surface functionalization, and strong interfacial regulation capability, which enable enhanced barrier properties, densification, and multifunctional protection of coatings. However, existing reviews have largely focused on the application of 2D carbon nanomaterials in anti-corrosive coatings, with a lack of systematic summaries on 0D carbon nanomaterials, particularly comprehensive reviews that combine quantitative bibliometric analysis with qualitative content analysis. To address this gap, this review employs a combined approach of bibliometric analysis and content analysis to systematically summarize the research progress of three typical types of 0D carbon nanomaterials, including nanodiamonds, fullerenes, and carbon dots, in the field of corrosion protective coatings. The quantitative analysis is conducted using CiteSpace 6.4 R.2 to reveal publication trends, research hotspots, and frontier evolution in this field, while the qualitative analysis selects representative studies to summarize application systems, performance characteristics, and underlying mechanisms. On this basis, the key challenges currently faced are identified, and future research directions are proposed. This review provides a systematic reference for the material design, mechanistic understanding, and engineering application of 0D carbon nanomaterial-based anti-corrosive coatings.

## 1. Introduction

Corrosion is a pervasive and costly challenge that affects virtually all sectors of the global economy, from infrastructure and transportation to energy and manufacturing [1,2,3]. The economic impact of corrosion is staggering: according to a US survey, corrosion costs six cents for every dollar of gross domestic product in the United States. Globally, this amounts to more than US$4 trillion per year [4]. In China alone, the total corrosion cost in 2014 was estimated to be CNY 2127.82 billion, accounting for approximately 3.34% of the national GDP [5]. Beyond these immense economic losses, corrosion poses significant threats to public safety and environmental integrity [6,7], leading to catastrophic failures of pipelines, bridges, and industrial equipment, as well as the release of hazardous materials. The urgent need for effective and reliable corrosion control strategies is therefore self-evident.

A variety of strategies have been developed to mitigate corrosion, including the use of corrosion inhibitors [8,9], cathodic protection [10,11], chloride ion solidification technologies [12,13], and protective coatings [14,15]. Among these, protective coatings are the most widely employed method due to their cost-effectiveness, ease of application, and ability to provide a physical barrier between the metallic substrate and the aggressive environment [14,15]. Organic coatings, such as epoxies, polyurethanes, and alkyds, dominate the market. However, conventional organic coatings possess inherent limitations. During curing, they often develop micro-pores and defects that serve as pathways for corrosive species. Furthermore, they are susceptible to aging, degradation under ultraviolet (UV) radiation, and mechanical damage (e.g., scratches and impact), all of which compromise their long-term protective performance.

**Figure 1:**
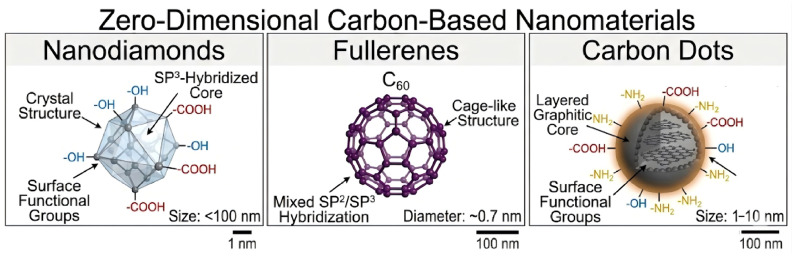
0D carbon-based nanomaterials are generally divided into three primary categories: ND, fullerenes and CDs. In this figure, blue represents hydroxyl groups (-OH), red represents carboxyl groups (-COOH), and yellow represents amino groups (-NH_2_).

Fortunately, the advent of nanotechnology has opened new avenues for enhancing the performance of protective coatings [16,17,18,19]. Among the various nanomaterials, zero-dimensional (0D) carbon-based nanomaterials have garnered significant attention due to their unique combination of properties. These materials, which include nanodiamonds (ND), fullerenes, and carbon dots (CDs) (Figure 1), exhibit ultra-small sizes, exceptionally high specific surface areas, quantum size effects, and versatile surface chemistry that facilitates functionalization [20,21,22]. Compared to their one-dimensional (1D), two-dimensional (2D), or three-dimensional (3D) counterparts, 0D carbon nanomaterials offer distinct advantages in coating applications. Their spherical or quasi-spherical morphology allows for effective filling of micro-defects, while their surface functional groups can be tailored to enhance compatibility with the matrix or to serve as reservoirs for corrosion inhibitors. Consequently, the application of these 0D carbon materials in corrosion protection, especially within advanced coating systems, has become a rapidly growing field of research.

While numerous review articles have summarized the application of carbon-based nanomaterials in corrosion protection, the vast majority have focused predominantly on 2D graphene and its derivatives [23,24,25,26]. This intense focus on 2D materials has resulted in a relative neglect of the unique potential offered by 0D carbon nanomaterials. A systematic and comprehensive review that critically evaluates the application effects, underlying mechanisms, and structure-property relationships of 0D carbon materials in anti-corrosive coatings is conspicuously absent. This gap in the literature may lead to an incomplete understanding of the field and may overlook promising research avenues. Furthermore, existing reviews often rely on a single methodology [23,24,25,26], either quantitative bibliometric analysis or qualitative content analysis, which can provide a one-sided perspective. Bibliometric analyses can reveal publication trends and research hotspots but often lack mechanistic depth, while purely qualitative reviews may be subjective and fail to capture the broader landscape of the field.

To address these gaps, this review employs a combined quantitative and qualitative approach to comprehensively evaluate the application and mechanisms of 0D carbon nanomaterials in anti-corrosive coatings. Using CiteSpace software, we conduct a bibliometric analysis to quantitatively map publication trends and growth patterns, as well as key research clusters and co-occurrence analysis. This quantitative foundation is then integrated with a detailed, qualitative, content-based analysis. The core of the review is structured by material type: Section 3 focuses on the application effects and anti-corrosion mechanisms of nanodiamonds (ND) in coatings; Section 4 examines fullerenes; and Section 5 reviews the rapidly developing field of carbon dots (CDs). Finally, Chapter 6 summarizes the current state of research, identifies key challenges, and proposes future directions for the development of high-performance 0D carbon-based anti-corrosive coatings.

## 2. Quantitative Analysis: A Bibliometric Perspective

### 2.1. Data Sources and Methodology

To comprehensively cover the research on 0D carbon-based nanomaterials in the field of anti-corrosive coatings and to minimize potential retrieval bias associated with a single database, this review selected the Web of Science Core Collection (WoSCC) as the literature data source. This database offers strong disciplinary coverage and high data quality, providing relatively complete bibliographic information, abstracts, keywords, and citation data for bibliometric analysis [27,28]. The literature search was completed on 31 March 2026. To systematically retrieve research on the application of 0D carbon-based nanomaterials, including fullerenes, CDs, and ND, in anti-corrosive coatings, a search strategy was constructed around three dimensions: material type, coating system, and corrosion protection. The search query employed Boolean logic operators, as detailed below: (“fullerene*” OR “carbon dot*” OR CDs OR C-dot* OR “carbon nanodot*” OR “graphene quantum dot*” OR CQD* OR “carbon nanoparticle*” OR “polymer dot*” OR “carbonized polymer dot*” OR “carbon quantum dot*” OR “nanodiamond*” OR “zero-dimensional carbon nanomaterial*”) AND (coat*) AND (corrosion OR anticorrosion OR inhibit*). The wildcard character “*” was used to cover different variants of terms, enhancing the comprehensiveness and inclusivity of the search results.

To ensure data relevance and consistency, only research articles published in English journals were retained, while non-research literature such as reviews, conference abstracts, editorials, book chapters, patents, and errata were excluded. Subsequently, the records exported from WoSCC were merged, deduplicated, and preliminarily screened to remove duplicate records and those with low relevance to the topic. The final retained literature included complete bibliographic information such as titles, abstracts, keywords, and references, forming the original database for subsequent bibliometric analysis. A total of 572 articles were included in the final analysis. This review employed CiteSpace (Version 7.0.0) as the primary bibliometric analysis tool to perform visual analysis of the knowledge structure and evolutionary trends in this field. The analysis mainly included annual publication trends, keyword co-occurrence analysis, and burst detection, aiming to reveal the research hotspots, evolutionary paths, and frontier directions of 0D carbon-based nanomaterials in the field of anti-corrosive coatings. Consistent with common settings in existing bibliometric studies, the time slice was set to 1 year, and network pruning was performed using the Pathfinder algorithm to highlight key structural relationships and improve map readability [29]. In the keyword analysis, the top 10% of nodes by frequency were selected for each time slice, with a maximum node count per slice not exceeding 100, balancing information completeness and map clarity [30]. The methodology described above provides a unified data foundation and technical support for the subsequent quantitative analysis.

In this study, WoSCC was selected as the sole data source because CiteSpace provides stable support for standardized WoSCC records, including cited references, author keywords, abstracts, and citation information. The use of a single database also helped maintain data consistency and avoid duplication or format incompatibility during bibliometric processing. However, this database selection may introduce indexing bias, since some regional journals, Asian publications, and conference proceedings may be covered differently in Scopus, Dimensions, or other databases. Therefore, the bibliometric results should be interpreted within the scope of the WoSCC dataset. Future studies could further integrate multiple databases and apply more systematic deduplication and normalization procedures to improve the comprehensiveness of the analysis.

It should be noted that the performance values summarized in this review were obtained under different experimental conditions; therefore, direct numerical comparison across unrelated systems should be made with caution and preferably within similar coating families and test protocols.

### 2.2. Publication Trends and Growth Patterns

**Figure 2:**
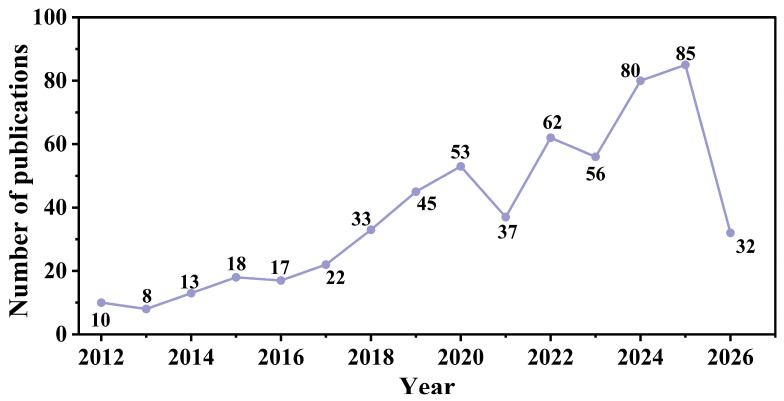
Annual publication trend chart.

As shown in Figure 2, the number of publications on 0D carbon-based nanomaterials in the field of anti-corrosive coatings generally exhibited a fluctuating upward trend from 2012 to 2025, increasing from 10 articles in 2012 to 85 articles in 2025. This indicates that this research direction has gradually evolved from early exploration into a significant branch of anti-corrosion coating research. Although slight fluctuations occurred in 2013, 2016, 2021, and 2023, the overall growth trend is evident. Notably, the increase in publications became more pronounced after 2018, suggesting a sustained rise in research interest in this field. Data for 2026 includes only 32 articles, primarily because the data for this year is not yet fully indexed, making it not strictly comparable.

From a stage perspective, the period from 2012 to 2017 represents an initial exploratory stage, with annual publications ranging from 8 to 22, indicating a relatively small overall scale. Research during this period primarily focused on the preliminary application of materials such as fullerenes, CDs, and ND in anti-corrosive coatings. The period from 2018 to 2020 marked a rapid growth stage, with publications increasing from 33 to 53, suggesting a shift towards more in-depth or broader research content. The temporary decline in publications to 37 in 2021 can be viewed as a phase of adjustment. From 2022 to 2025, the field entered a highly active development stage, with publications reaching 62, 56, 80, and 85, respectively, maintaining high growth overall. This indicates that 0D carbon-based nanomaterials have become a research hotspot in the field of anti-corrosion protection.

### 2.3. Key Research Clusters and Co-Occurrence Analysis

**Figure 3:**
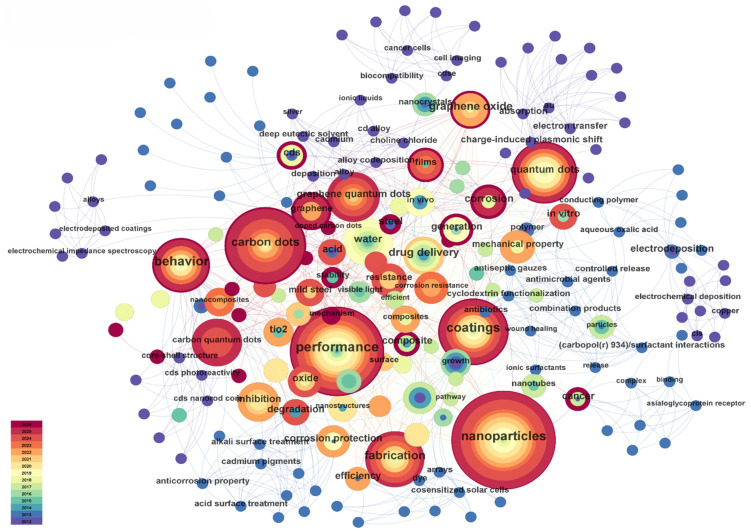
Keyword co-occurrence map.

Keyword co-occurrence analysis intuitively reflects the research hotspots and their intrinsic connections in the field of 0D carbon-based nanomaterials for anti-corrosion applications. As shown in Figure 3, high-frequency keywords in the network include nanoparticles (103), performance (75), carbon dots (60), coatings (47), quantum dots (40), behavior (33), and fabrication (33). This indicates that research in this field primarily revolves around the introduction of nanoparticles, enhancement of coating performance, application of CDs/quantum dot materials, and their preparation and functional behavior. Among these, nanoparticles, performance, and coatings not only appear with high frequency but also exhibit high centrality (0.29, 0.25, and 0.24, respectively), suggesting that these keywords act as crucial hubs within the research network, connecting material design, coating construction, and performance evaluation.

Examining the keyword distribution, the core research directions in this field mainly focus on the following aspects. First, the enhancement of overall coating performance by 0D carbon-based nanomaterials is a central theme, represented by keywords such as performance, coatings, and behavior. Research in this direction focuses on the impact of material addition on corrosion resistance, barrier properties, mechanical properties, and service behavior. Second, research on CDs is a highly active branch, represented by keywords such as carbon dots, quantum dots, graphene quantum dots (GQDs), and carbon quantum dots (CQDs). This indicates that CDs, especially their subcategories (GQDs and CQDs [31]), have become the most active research branch in this field. Third, the direction of preparation and fabrication, represented by keywords such as fabrication, electrodeposition, nanocrystals, and particles, reflects the growing emphasis on how the introduction methods, dispersion state, and film formation processes of 0D carbon materials in different coating systems affect anti-corrosion performance. Finally, keywords such as water, behavior, and generation indicate that research often focuses on corrosion behavior in aqueous environments, interfacial reaction processes, and protection mechanism analysis.

Overall, the keyword co-occurrence results show that the main research line for 0D carbon-based nanomaterials in the anti-corrosion field is relatively clear, progressively unfolding around material design, coating construction, performance enhancement, and mechanism analysis. Among these, CDs have emerged as the most representative current research hotspot, while coating performance optimization and preparation method regulation constitute two important mainstays for the sustained development of the field.

### 2.4. Evolution of Research Frontiers

Burst detection analysis effectively reflects the shift in research hotspots and the evolution of frontier directions over different periods. As shown in Table 1, from 2012 to 2026, the research hotspots for 0D carbon-based nanomaterials in the anti-corrosion field exhibited a trend of evolution, progressing from early thin-film construction and particle application, towards performance enhancement and surface regulation, and further towards composite anti-corrosion systems dominated by CDs. Within the 2012–2026 statistical window of this table, the red blocks indicate the period of significant citation burst for the corresponding keyword, while the light gray and cyan blocks indicate the non-burst period.

In the early stage (2012–2018), prominent burst keywords included thin films, particles, and water. Among these, thin films had the earliest burst and lasted for a relatively long period, indicating that early research focused on the preliminary application of 0D carbon materials, such as fullerenes and ND in thin films or surface overlayers. The emergence of particles reflected that nanoparticle filling, dispersion, and their basic protective effects were key concerns during this period. The burst of water indicated that related research often focused on corrosion behavior and protective effectiveness in aqueous environments.

In the middle stage (2018–2023), the burst keywords gradually shifted towards mechanical property, graphene oxide, carbon nanotubes, inhibition, resistance, enhancement, surface modification, shell, efficiency, nanocomposites, and corrosion resistance. This stage indicates that the research focus transitioned from simply introducing materials to optimizing performance and deepening mechanistic understanding, with particular emphasis on coating mechanical properties, corrosion resistance, and surface modification strategies. Notably, although graphene oxide and carbon nanotubes are not 0D carbon materials, their burst suggests that during this period, research commonly incorporated comparisons or synergistic design concepts with 1D and 2D carbon materials, thereby promoting the development of 0D material anti-corrosion systems towards composite approaches.

In the recent stage (2024–2026), the keywords with the highest burst strength are carbon quantum dots (8.34) and carbon dots (7.39), significantly higher than other burst terms. This indicates that CQDs and CDs have become the most active and representative current frontiers. Concurrently, composite coatings and mild steel also exhibited bursts during this period, suggesting that current research is increasingly focused on the application of CD-based materials in composite anti-corrosive coatings, gradually moving towards practical metal substrates, especially for the protection of mild steel.

It should be noted that the burst strengths of “carbon quantum dots” and “carbon dots” were generated by CiteSpace based on the retrieved WoSCC dataset and were not manually adjusted. These values indicate that CD-related terms showed a strong increase in the present dataset during 2024–2026. However, they should not be directly compared with burst values from other research fields, because burst strength can be affected by dataset size, search strategy, time span, and keyword normalization. In addition, the recent burst of CD-related keywords may partly reflect the evolution of terminology, as some authors have gradually replaced broader expressions such as “quantum dots” with more specific terms such as “carbon dots” and “carbon quantum dots.” Therefore, these burst results should be interpreted as evidence of an increasing research focus on CD-based anti-corrosive coatings within the retrieved dataset, rather than as an absolute cross-field metric.

Overall, the evolution of burst keywords clearly demonstrates that the main research line in this field has progressed from the initial exploration of films/particles, through performance enhancement and surface functionalization regulation, towards the current focus on composite anti-corrosive coatings and engineering applications centered on CD-based materials. This also indicates that CQDs and related CDs are becoming the key driving force behind the sustained growth of research on 0D carbon-based nanomaterials for anti-corrosion applications.

## 3. ND in Anti-Corrosive Coatings

ND are diamond carbon particles typically ranging from a few to several tens of nanometers in size, commonly produced by the detonation method [32]. Their typical structure can be summarized as a “diamond core-transitional carbon layer-surface functional layer”: the core is primarily composed of sp^3^ carbon, imparting ultra-high hardness, wear resistance, and high thermal conductivity; the surface is rich in active groups such as hydroxyl and carboxyl groups, facilitating further oxidation, hydroxylation, amination, and silanization modification. Consequently, ND exhibit excellent mechanical properties, chemical inertness, electrical insulation, biocompatibility, and functionalizability. Based on these properties, ND have been widely used in fields such as wear protection, biomedical applications, sensing, energy, and composite materials [33,34,35,36]. In anti-corrosive coatings, their value is primarily reflected in defect filling, densification enhancement, wear-corrosion synergy, prevention of galvanic corrosion induced by conductive fillers, and serving as carriers for corrosion inhibitors or interface-active components.

### 3.1. Application Performance of ND-Based Coatings

The application of ND in the field of anti-corrosive coatings has gradually evolved from simple surface coverage to multi-type, multi-scenario composite protection systems. Current research mainly focuses on four typical systems: standalone films or diamond-like carbon (DLC)-related films, metal matrix composite coatings, organic resin-based composite coatings, and functional protective layers such as sol–gel and superhydrophobic coatings. Meanwhile, their application boundaries have extended from the surface protection of traditional engineering structural materials to emerging fields such as flexible conductive films and aqueous metal electrode interfaces. Therefore, it is necessary to classify and summarize the application of ND according to different coating systems to more clearly outline their application characteristics and development trends in anti-corrosive coatings.

**Figure 4:**
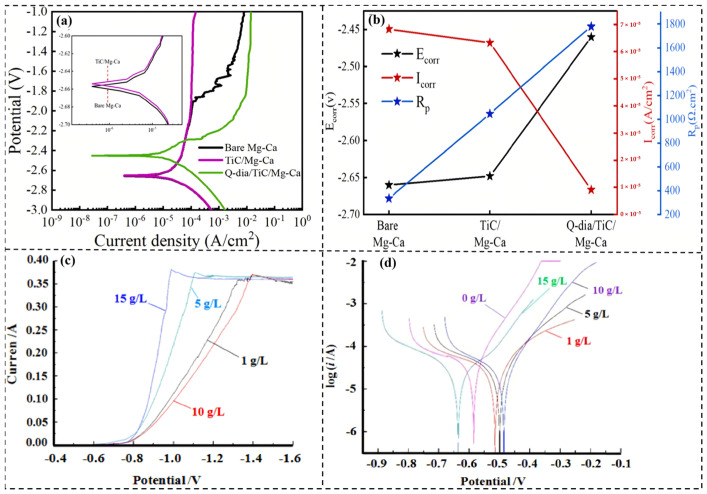
Electrochemical and Mechanical Characterization of Different Carbon-Based Protective Coatings. (**a**) Potentiodynamic polarization curves of the Mg-Ca alloy and composite coatings [40]; (**b**) Comparison of corrosion parameters for the corresponding samples [40]; (**c**) Cathodic polarization behavior of Ni ions in plating solutions with different nanodiamond (ND) contents [43]; (**d**) Tafel polarization curves of Ni-ND composite coatings with varying ND concentration [43].

In terms of standalone films, Bhattacherjee et al. [37] deposited DLC and nitrogenated DLC (N-DLC) films on Ti-6Al-4V and found that direct deposition of DLC was prone to early delamination due to internal stress, whereas pre-depositing ND particles significantly improved adhesion, resulting in denser, smoother, and more uniform films. The average hardness of DLC was about 11 GPa, while that of N-DLC was about 9 GPa, but nitrogen doping further enhanced adhesion and corrosion resistance, indicating that ND primarily serves as an “interface seed/adhesion-promoting layer” rather than merely enhancing hardness. Subsequently, Oraby et al. [38] prepared Q-dia diamond coatings on Mg-Ca alloys and found that a TiC interlayer was most conducive to obtaining uniform, dense films without obvious defects, increasing the polarization resistance from 0.336 kΩ·cm^2^ to 1.78 kΩ·cm^2^ and reducing the corrosion current density to 9.18 × 10^−6^ A·cm^−2^. This demonstrated that dense sp^2^/sp^3^ coexisting carbon layers can significantly improve the corrosion resistance of magnesium alloys in NaCl. Furthermore, Aramesh et al. [39] constructed a hybrid diamond/amorphous DLC ultra-thin protective layer on the surface and within the pores of nanoporous anodic alumina, resulting in nanostructures with excellent chemical and corrosion stability, indicating that ND-related carbon layers are also suitable for protecting inorganic nanostructured surfaces. For cemented carbide substrates, Mohamed et al. [40] (Figure 4a,b) used coaxial arc plasma deposition to obtain ND composite thick films of approximately 16 μm thickness. At 7.0 J/pulse, the hardness reached 72.5 GPa, the friction coefficient was as low as 0.09, and the corrosion rate decreased to 0.5136 mpy, representing a 60.4% improvement over uncoated samples. This demonstrates the potential of ND thick films to simultaneously provide wear and corrosion resistance for cutting tools and cemented carbide surfaces.

In metal matrix composite coatings, ND applications have expanded to various systems such as Ni-P, Ni-B, Ni, Al, Ag, Ti, Cr, and NiAl [41,42,43,44,45,46,47,48,49,50,51,52]. Their common feature is the incorporation of ND into the metal matrix through methods such as electroless plating, electrodeposition, flame spraying, plasma spraying, or layer-by-layer spin coating, thereby improving coating densification, surface morphology, grain structure, and overall protective performance (Figure 4c,d). For instance, electroless Ni-P/ND plating exhibited optimal performance at 1 g/L ND concentration, with microhardness increasing from 608 Hv to 957 Hv and corrosion current density decreasing from 0.416 to 0.152, indicating that ND can effectively improve the densification and corrosion resistance of Ni-P coatings [41]. For Ni-ND systems, both pulsed electrodeposition and room-temperature tartrate electrodeposition studies have shown that an appropriate amount of ND can shift the corrosion potential positively, reduce the corrosion current, and increase polarization/charge transfer resistance. At an ND concentration of 5 × 10^−2^ g·dm^−3^, the charge transfer resistance reached 283.99 kΩ·cm^2^ [50,52]. Additionally, Ag/ND composite films exhibited a polarization resistance increased to 2.213 × 10^6^ Ω·cm^2^, approximately 20 times that of pure Ag films, while also maintaining improved electrical conductivity [47]. In thermal spray systems, Ti–ND coatings at 0.1 wt.% ND reduced the corrosion rate from 5.89 to 3.162 mpy, but further increasing the content led to a decrease in corrosion resistance due to micro-galvanic effects [42]. For the NiAl system, 0.1 wt.% ND reduced the corrosion rate to 0.16 mpy [49], while 1 wt.% ND significantly reduced high-temperature hot corrosion weight gain [45]. Other systems, such as NiB-ND, Cr–ND, and Al–ND have also generally exhibited trends of reduced porosity, denser films, and enhanced corrosion protection [44,46,48].

In organic coatings, the advantages of ND derive more from dispersion and interface control. Sorkhabi et al. [53] incorporated ND into polyaniline (PANI) coatings, achieving a corrosion protection efficiency of approximately 90% for low-carbon steel after 3 days. The corrosion current density decreased from 4.22 × 10^−6^ to 1.35 × 10^−6^ A·cm^−2^, and the coating porosity was reduced by nearly 18 times compared to pure PANI, demonstrating that ND can promote the formation of denser, low-porosity colloidal films of conductive polymers. Rahmani et al. [54] further loaded thermally oxidized ND with dodecylamine as a corrosion inhibitor and introduced them into epoxy. After 30 days of immersion in 3.5 wt.% NaCl, the low-frequency impedance of the 1 wt.% DND/EP coating was at least one order of magnitude higher than that of OND/EP and pure EP, indicating that ND can serve not only as barrier fillers but also as pH-responsive nanocarriers for corrosion inhibitors. Pan et al. [17] achieved monodispersion of ND through strong OH^−^ shear treatment, increasing the absolute zeta potential to −54.2 mV and maintaining stable dispersion for 200 days. After incorporation into waterborne epoxy (WEP), the |Z|_f_ = 0.01 Hz of the m-NDs/WEP coating was nearly three orders of magnitude higher than that of pure WEP after 168 h, with a more positive *E*_corr_ and *I*_corr_ reduced to 1.07 × 10^−9^ A·cm^−2^. Furthermore, Xu et al. [55] used organosilane-bridged ND to modify graphene oxide (GO) and constructed a composite anti-corrosion coating with vinyl ester resin. The conductivity of the modified GO decreased to 9.6 × 10^−9^ S/cm, and after 120 days of immersion in 3.5 wt.% NaCl, the |Z|_0.01Hz_ remained at 1.00 × 10^9^ Ω·cm^2^, with a protection efficiency of 97.8%, demonstrating a clear synergistic anti-corrosion effect between ND and 2D GO. Recent studies have also shown that SiO_2_@ND hybrid particles can simultaneously endow epoxy with “barrier + self-healing” dual functions [56]. At an addition level of 0.33 wt.%, the corrosion protection enhancement efficiency for scratched coatings reached 138%, and intact coatings maintained higher low-frequency impedance after 22 weeks of immersion.

In sol–gel and superhydrophobic systems, ND also exhibits outstanding performance. Nezamdoust et al. [57] introduced hydroxylated ND (HND) into a sol–gel film on magnesium alloys and found that the optimal corrosion resistance was achieved at 0.01 wt.%, with micron-scale defects disappearing, indicating that HND can improve film densification and fill defects through chemical interaction with the silane network. Uzoma et al. [58] prepared a superhydrophobic ND/organic resin coating with a thickness of only 10 μm, which exhibited an impedance on the order of 10^6^ Ω·cm^2^ after just 4 h of initial immersion, along with a contact angle of 154° and a sliding angle of only 6°. Another study reported a root-like penetrating Ep-FND-S coating that reduced the rapid chloride ion migration charge to 264 C, significantly lower than the 3600 C of uncoated mortar, demonstrating that ND can enhance long-term water resistance by constructing rough hydrophobic surfaces and air layers [59].

Recent studies have further extended the application of ND to emerging coating-related protection scenarios, particularly in the form of interfacial/protective layers on electrochemical substrates. Liu et al. [60] constructed an ND interfacial layer on the Zn anode surface, establishing nucleation sites on the order of ~10^12^ cm^−2^ due to the ultra-high surface energy, enabling Zn symmetric cells to operate stably for over 3600 h. Ma et al. [61] incorporated ND into a sodium alginate hydrogel to form a protective layer for Zn powder anodes, achieving stable cycling for 3780 h at 5 mA·cm^−2^, a 126-fold improvement over bare Zn powder. This indicates that ND has expanded from traditional “structural anti-corrosive coatings” to a broader concept of “electrochemical interface protective layers.”

### 3.2. Anti-Corrosion Mechanism of ND-Based Coatings

The mechanism of ND in anti-corrosive coatings is not a single “filler reinforcement” effect but involves multiple aspects such as barrier shielding, interfacial densification, localized corrosion inhibition, active protection, and wear-corrosion synergy. Its effectiveness is influenced by substrate type, surface functionalization, dispersion state, and addition level.

The most common anti-corrosion mechanism of ND in coatings is physical barrier and defect filling [17,56,57]. ND particles, due to their small size and high specific surface area, can fill micropores, microcracks, and loose regions in organic coatings, metal composite coatings, and sol–gel films, thereby reducing the permeation pathways for water, oxygen, and Cl^−^ and prolonging diffusion paths. Taking the magnesium alloy sol–gel system as an example, the addition of hydroxylated ND significantly reduced micron-scale defects in the film, with optimal corrosion resistance at 0.01 wt.%; the authors explicitly attributed this to the denser film, more tortuous diffusion pathways, and defect filling by nanoparticles [57].

The performance enhancement of ND largely stems from interfacial chemical coupling and film densification [54,56,57]. Their surfaces are rich in active groups such as hydroxyl and carboxyl groups, which, after hydroxylation, amination, or silanization, can form stronger interfacial interactions with epoxy resins or silane precursors. In sol–gel systems, the chemical interaction between HND and the silane network promotes the formation of a more compact three-dimensional structure. In epoxy, DDA-modified DND, due to more uniform dispersion and stronger compatibility with the resin, exhibited low-frequency impedance at least one order of magnitude higher than that of OND/EP and pure EP after 30 days [56]. Similarly, FSiO_2_@sND hybrid particles reduced the hydrophilicity of the hybrid particles and increased crosslinking density and coating/substrate adhesion through the carboxyl groups on ND interacting with APTES [56].

ND also exhibits an important localized corrosion inhibition effect, particularly in suppressing pit propagation [51]. Studies on Ni-B-ND coatings have shown that ND addition did not significantly alter the pit nucleation rate but effectively prevented further pit growth. SEM observations revealed stable pitting on the Ni-B surface, while the Ni-B-ND surface showed virtually no localized corrosion. Molecular dynamics simulations further demonstrated that Cl^−^ has lower adsorption energy and weaker chemical interaction on ND surfaces, making it less likely to accumulate around ND, thereby inhibiting stable pit formation [51].

In organic systems, ND can also serve as nanocarriers for corrosion inhibitors and pH-responsive self-healing units [56]. The FSiO_2_@sND system reported by Dabaleh et al. [56] demonstrated that APTES could be released under alkaline conditions and act as a corrosion inhibitor, while the carboxyl groups on the ND surface could participate in forming a stable protective film. At 0.33 wt.% addition, the corrosion protection enhancement efficiency for scratched coatings reached 138%, and intact coatings maintained higher low-frequency impedance after 22 weeks, reflecting a dual “barrier + self-healing” protection mechanism.

ND also exhibits notable insulation regulation and synergistic protection effects [55]. When combined with 2D materials, they not only provide 0D filling but also reduce the risk of galvanic corrosion induced by conductive fillers. Related studies have shown that when ND synergize with GO, they can combine the barrier effect of 2D sheets with the defect-sealing capability of 0D particles, thereby constructing a more stable long-term protective network.

The wear-corrosion synergy mechanism of ND is equally critical [49,57]. Their sp^3^ diamond core provides ultra-high hardness and excellent wear resistance, maintaining coating integrity under friction, erosion, and mechanical impact conditions, and slowing crack initiation and propagation. Therefore, their contribution is reflected not only in improved electrochemical parameters under static immersion conditions but also in long-term structural stability under complex service environments.

In summary, the core advantages of ND in anti-corrosive coatings lie in their ability to both fill defects and prolong diffusion pathways through their 0D structure and achieve interfacial reinforcement, localized corrosion inhibition, and active protection coupling through surface designability. Hence, their value is not merely “hardness” but rather their multifunctional synergistic protection potential. It is important to note that ND are not “the more, the better” [42,49]. Excessive addition can easily lead to re-agglomeration, local defects, and interfacial discontinuity, and may even induce micro-galvanic corrosion, resulting in decreased corrosion resistance. In sol–gel, Ti-ND, and NiAl-ND systems, the phenomenon of optimal performance at low addition levels and deterioration at high addition levels has been consistently observed.

## 4. Fullerene in Anti-Corrosive Coatings

Fullerene is a 0D carbon allotrope, first discovered by Kroto, Smalley, and Curl in 1985 [62]. The most abundant and stable member of this family, fullerene C60, possesses a unique hollow cage-like structure composed of 20 hexagons and 12 pentagons, with all carbon atoms being sp^2^-hybridized. This distinctive molecular architecture endows C60 with a series of remarkable physicochemical properties, including high electron affinity, excellent free radical scavenging capacity (often termed a “free radical sponge”), intrinsic hydrophobicity, and outstanding thermal and chemical stability. These characteristics make fullerene a highly promising functional nanomaterial, with applications spanning industrial lubricants, medical therapeutics, energy storage devices, and, as the focus of this review, protective coatings [63,64,65,66,67]. In the field of anti-corrosive coatings, fullerene can act as a physical barrier and a nanofiller to enhance coating densification, thereby improving corrosion protection performance while simultaneously enhancing the mechanical and tribological properties of the coating.

### 4.1. Application Performance of Fullerene-Based Coatings

Significant progress has been made in the application of fullerenes for corrosion protection, evolving from simple physical vapor-deposited films to functionalized nanocomposite systems successfully integrated into both organic and inorganic matrices. Current research primarily focuses on four typical systems: standalone films or fullerene-like carbon-related films, metal matrix composite coatings, organic resin-based composite coatings, and functional protective layers such as sol–gel coatings. Concurrently, their application scope has expanded from the surface protection of traditional engineering structural materials to emerging electrochemical interfaces, such as aqueous metal electrodes. This evolution indicates that the role of fullerenes in corrosion protection is no longer limited to “0D fillers” but is progressively shifting towards composite protective units that combine structural regulation and interface modulation.

Early research focused on the direct application of fullerenes as physical barriers. Sittner et al. [68] deposited fullerene (C60/C70 mixture) films onto pure iron substrates via thermal evaporation and found that the as-deposited 180 nm thick fullerene film reduced the anodic dissolution current density by approximately one order of magnitude. However, its performance was limited by inherent porosity and weak intermolecular interactions. To overcome this limitation, the researchers modified the coating using Ar^+^ ion irradiation (2 keV, fluence of 2 × 10^17^ cm^−2^). This treatment reduced the critical current density for iron dissolution by two orders of magnitude, and Raman spectroscopy revealed that the ion irradiation transformed the molecular fullerene film into a dense amorphous carbon network, effectively sealing pinholes. In contrast, thermal annealing at 1200 K, while also converting fullerenes to amorphous carbon, resulted in a corrosion rate even higher than that of uncoated iron, attributed to the formation of iron carbide (Fe_3_C) at the carbon/iron interface, which induced galvanic corrosion. Subsequently, Sittner and Ensinger [69] systematically characterized the porosity of fullerene films using cyclic voltammetry, introducing two complementary electrochemical metrics: the critical current density (*I*_crit_) and the open circuit potential (OCP) shift. As the ion fluence increased to 1 × 10^17^ cm^−2^, *I*_crit_ remained stable while the OCP shifted positively, indicating that the high-energy ions primarily densified and narrowed the pores in the upper region of the film, creating a more tortuous path for electrolyte diffusion.

Incorporating fullerenes as a dispersed phase into electrodeposited metal matrices represents an effective strategy to combine the structural integrity of metals with the functional properties of C60. Tseluikin and co-workers conducted a series of studies on nickel-fullerene C60 composite electrodeposited coatings (CECs) [70,71]. Adding a stable aqueous C60 suspension (average particle size 24 nm) to a nickel-plating bath facilitated the cathodic process, allowing deposition at less negative potentials. Secondary ion mass spectrometry (SIMS) analysis confirmed the presence of C-H bonds, indicating cathodic hydrogenation of fullerenes during co-deposition [71]. A subsequent study in 2017 quantified the advantages of these composite coatings: the sliding friction coefficient was reduced by a factor of 1.8 to 2.5 (attributed to the solid lubrication effect of fullerenes), and they exhibited a wider passive region and lower anodic dissolution current in 0.5 M H_2_SO_4_ [70]. Mohammadpour and Zare [72] extended this concept to a Ni-W ternary alloy system, comparing the shape effect of 0D fullerenes with 1D (SWCNTs, MWCNTs) and 2D (graphene oxide nanosheets, GONSs) fillers. The results showed that Ni-W coatings reinforced with asymmetric fillers (MWCNTs, GONSs) were denser and had fewer cracks. Electrochemical impedance spectroscopy (EIS) revealed that these coatings exhibited the highest polarization resistance (*R*_p_) and the lowest corrosion current density (*j*_corr_) (for Ni-W/GONS, *j*_corr_ = 2.66 μA cm^−2^, inhibition efficiency 90.84%; whereas for Ni-W/C60, *j*_corr_ = 27.91 μA cm^−2^, inhibition efficiency only 4.03%), confirming the advantages of sheet-like and high-aspect-ratio fillers in filling micropores and creating tortuous paths.

In the realm of advanced inorganic coatings, Dwivedi et al. [73] employed a filtered cathodic vacuum arc (FCVA) process with a dual-energy surface modification (first 350 eV, then 90 eV C^+^ ions) to develop an ultra-thin (~1.7 nm) carbon overcoat (COC) for high-density magnetic storage media. High-resolution transmission electron microscopy and scanning tunneling microscopy revealed that the coating consisted of an amorphous carbon matrix with interspersed graphene and fullerene-like nanostructures. Although 37% thinner than the commercial COC (~2.7 nm), it exhibited a lower coefficient of friction (~0.25), higher wear resistance, and comparable oxidation/corrosion resistance. The performance enhancement was attributed to the higher sp^3^ carbon bond content (40% vs. 33%), the defect-sealing effect of the unique nanostructures, and the lower surface roughness.

**Figure 5:**
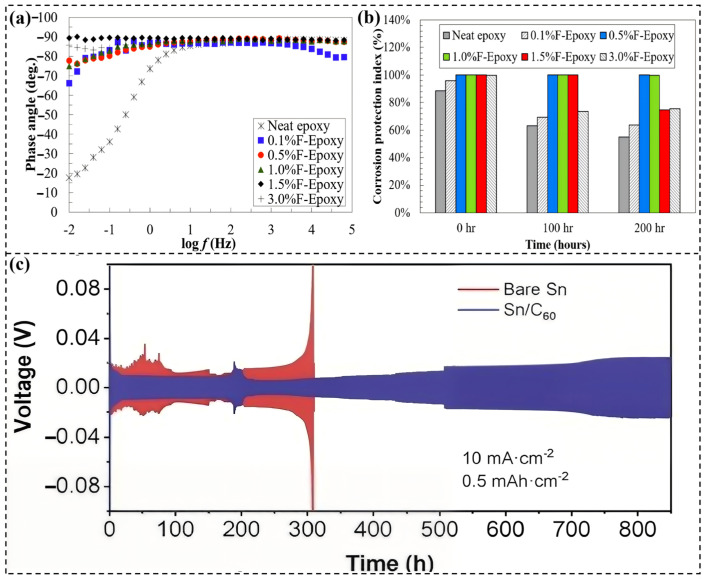
(**a**) Bode phase angle plots of C60 -modified epoxy coatings with various loadings. The neat epoxy coating offers basic corrosion protection but fails to form an effective dense barrier, evidenced by a clear low-frequency inflection in the impedance curve [75]. (**b**) Corrosion protection index of coatings at different immersion times, consistent with Bode results. C60 significantly enhances coating corrosion resistance and stability, with 0.5 and 1.0 wt.% C60 -modified coatings remaining fully intact to block corrosive media penetration during immersion [75]. (**c**) Sn stripping/plating voltage-time profiles for bare Sn and Sn/C60 electrodes at 10 mA·cm^−2^ and 0.5 mAh·cm^−2^, showing Sn stripping/plating behavior on C60 coatings; Sn(OH)_3_^−^ ions are randomly dispersed via Brownian motion in the initial alkaline electrolyte [79].

To fully leverage the synergistic effects of fullerenes with polymers, numerous studies have incorporated them into organic coatings, particularly epoxy resins. Liu et al. [74] chemically functionalized C60 with a silane coupling agent (KH550) and incorporated it into an epoxy matrix at an optimal loading of 0.5 wt.%. The functionalized C60 (FC60)/epoxy coating exhibited a lower friction coefficient and wear area, as well as higher corrosion resistance compared to pure epoxy. Compared to functionalized graphene (FG)/epoxy coatings, FC60/epoxy showed better tribological properties, while FG/epoxy exhibited superior corrosion resistance. This was attributed to the difference in filler shape: the spherical C60 acts as a nanobearing, while the sheet-like graphene creates a more effective labyrinth effect. Wang et al. [75] developed a solvent-free dispersion method to effectively disperse unmodified C60 in epoxy for pipeline coatings. Coatings containing 0.5 and 1.0 wt.% C60 remained intact after 200 h of salt spray exposure, with a coating corrosion protection index (CCPI) of 100%, whereas the performance of the pure epoxy coating decreased by more than 50% (Figure 5a,b).

In hybrid sol–gel coatings, fullerenes have demonstrated significant protective effects for reactive metals like magnesium alloys. Samadianfard et al. [76] incorporated oxidized fullerene (OF) nanoparticles into a TEOS-GPTMS sol–gel coating. FTIR confirmed chemical crosslinking between the carboxyl/hydroxyl groups on OF and the silanol groups (Si-OH) of the hydrolyzed silane precursors, resulting in a defect-free dense film. The surface roughness decreased from 67.5 nm to 40.3 nm, and the *R*_p_ increased significantly. Subsequently, the same team developed an intelligent active protection system [77]: they first aminated fullerenes (F-NH_2_) and then stabilized the corrosion inhibitor sodium dodecyl sulfate (SDS) onto the nanoparticles (F-SDS) before incorporating them into the sol–gel coating. The F-SDS coating exhibited the highest corrosion protection performance, attributed to a dual mechanism: the physical barrier effect of the nanoparticles and the pH-responsive release of SDS at the onset of corrosion, actively inhibiting the corrosion process.

Recent studies have also extended the application of fullerene-based protective coatings/layers to emerging battery and catalytic systems. Huang et al. [78] developed a three-dimensional epoxy coating (CUGC-250) combining modified carbon nanotubes, reduced graphene oxide, modified fullerenes, and a UV absorber, providing excellent corrosion and UV resistance for polycarbonate/acrylonitrile-styrene-acrylic substrates. Electrochemical tests showed that steel coated with CUGC-250 exhibited a corrosion current density as low as 0.335 μA cm^−2^ and a protection efficiency of 99.5%. Furthermore, the application of fullerenes has extended beyond traditional structural coatings to corrosion protection scenarios in batteries and catalysis. Wang et al. [79] applied a nanoscale C60 coating on a tin (Sn) metal anode using physical evaporation. This coating acted as an electric field regulator, promoting uniform Sn deposition and suppressing dendrite formation, enabling stable cycling of symmetric cells for over 850 h (Figure 5c). Song et al. [80] utilized fullerenes as precursors for carbon supports to prepare CoRu/CNB electrocatalysts. The fullerene-derived carbon layer endowed the catalyst with excellent resistance to both acidic and alkaline corrosion.

### 4.2. Anti-Corrosion Mechanism of Fullerene-Based Coatings

Based on the extensive literature reviewed, the anti-corrosion mechanisms of fullerene C60 and its composites can be summarized into the following key aspects.

Physical barrier effect is the most fundamental mechanism of fullerenes. Fullerene nanoparticles, whether as standalone films or as fillers, can block or prolong the diffusion paths of corrosive media (H_2_O, O_2_, Cl^−^). Ion beam treatment can transform porous fullerene films into dense amorphous carbon networks, effectively sealing pinholes and nanopores [68,69]. In composite coatings, well-dispersed C60 fills micropores and defects, forming a denser, less permeable coating, thereby delaying the penetration of corrosive media [71,75].

Formation of tortuous paths (labyrinth effect) is an important mechanism for enhancing barrier performance. When incorporated into a matrix, the 0D spherical C60 is less effective at creating tortuous paths compared to high-aspect-ratio 1D or 2D fillers. However, its unique geometry still contributes to increasing the diffusion path length. When combined with other nanomaterials (as in the CUGC-250 coating), fullerenes help form a complex multi-dimensional barrier network that significantly hinders electrolyte penetration, extending the path to the substrate [72,78].

Chemical interaction and defect repair mechanisms can enhance coating integrity and localized defect tolerance [76,77]. Functionalized fullerenes (e.g., oxidized or aminated) can form chemical bonds, achieving covalent bonding with the surrounding matrix. In sol–gel coatings, OF nanoparticles form Si-O-C and ester bonds with the silanol groups of the hydrolyzed silane precursors. This chemical crosslinking not only improves dispersion but also “repairs” inherent microcracks and defects within the brittle sol–gel network, resulting in a stronger, defect-free barrier.

Active corrosion inhibition mechanisms make fullerenes ideal carriers for smart coatings. Fullerenes can serve as excellent nanocarriers for corrosion inhibitors, enabling active response to corrosion. SDS molecules stabilized on F-SDS nanoparticles can be released in response to local pH changes at the onset of corrosion. The released SDS molecules then adsorb onto active cathodic sites on the metal, inhibiting the corrosion reaction. It should be noted that the pH-responsive release of SDS mainly provides inhibitor-triggered active protection, rather than complete structural self-healing of the coating [77].

Mechanical reinforcement and wear resistance mechanisms are important pathways for fullerenes to enhance coating durability. Due to its high compressive strength and spherical shape, fullerene can act as a “nanobearing” or toughening agent, effectively dispersing stress. This enhances the mechanical integrity, scratch resistance, and wear resistance of the coating [70,74]. Improved mechanical properties can prevent the formation of cracks, which are initiation points for corrosive media penetration, thus indirectly enhancing the coating’s corrosion protection performance.

**Figure 6:**
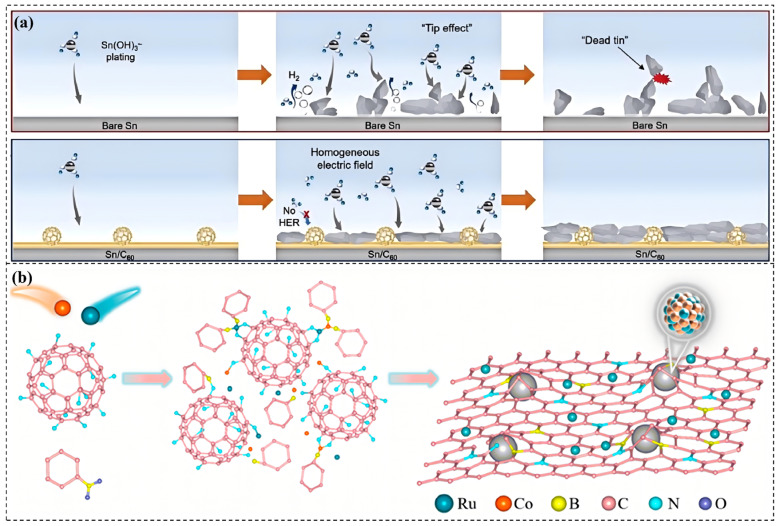
Schematic diagram of the dissolution/deposition behavior of tin on the C60-coated surface. (**a**) In the alkaline electrolyte system, Sn(OH)_3_^−^ ions are randomly dispersed in the initial state due to Brownian motion [79]; (**b**) Schematic diagram of the synthesis process and structure of the CoRu/CNB electrocatalyst. The Ru/CNB and Co/CNB electrocatalysts were prepared using the same procedure, except that CoCl_3_ and RuCl_3_ were not added, respectively [80].

Electric field regulation and dendrite suppression mechanisms extend the application of fullerenes in electrochemical systems. In battery applications, a C60 coating on a metal anode (e.g., Sn) can homogenize the surface electric field, ensuring a more uniform current distribution during metal electroplating. This mechanism prevents the sharp “tip effects” that lead to dendrite growth and “dead metal” formation. Suppressing uneven deposition is a crucial corrosion mitigation strategy in electrochemical systems, effectively prolonging battery cycle life (Figure 6a) [79].

The carbon layer-induced corrosion resistance mechanism gives fullerenes a unique advantage in catalysis. When fullerenes are used as precursors for carbon supports in catalysts, they can be pyrolyzed to form a thin, defect-rich, highly graphitized carbon layer. This carbon layer acts as a protective shell, encapsulating active metal nanoparticles (e.g., CoRu alloy), shielding them from direct contact with corrosive electrolytes (acidic or alkaline), thereby significantly enhancing the long-term operational stability of the catalyst (Figure 6b) [80].

In summary, the anti-corrosion mechanisms of fullerenes and their composites are not based on a single effect but result from the synergy of multiple mechanisms. Physical barrier and labyrinth effects provide passive protection by hindering the diffusion of corrosive media, delaying the onset of corrosion. Chemical interactions and defect repair enhance the structural integrity of the coating, eliminating potential weak points. Active corrosion inhibition mechanisms endow the coating with intelligent responsiveness, enabling active protection after damage. Meanwhile, mechanical reinforcement and electric field regulation improve the overall protective performance from the perspectives of structural strengthening and interface modulation. From the early single physical barrier to today’s multifunctional smart coatings, the application of fullerenes in corrosion protection is continuously evolving towards multifunctionality, intelligence, and the integration of structure and function.

## 5. CDs in Anti-Corrosive Coatings

CDs are a class of emerging 0D fluorescent carbon-based nanomaterials, typically defined as quasi-spherical nanoparticles with a size smaller than 10 nm that are primarily composed of carbon and exhibit excellent fluorescence properties. CDs were first discovered in 2004 during the electrophoretic purification of single-walled carbon nanotubes [81] and were officially named in 2006 [82]. An individual CD generally consists of a carbon core and surface groups. The carbon core may contain sp^2^ carbon structures, nitrogen-doped sp^2^ carbon structures, or structural units formed by s-triazine structures, benzene ring units, and aliphatic groups connected via C-N bonds; the surface is typically rich in oxygen- and nitrogen-containing groups [21]. Based on the carbon core structure and surface chemical state, CDs are generally classified into GQDs, CQDs, carbon nanodots (CNDs), and carbonized polymer dots (CPDs) [83]. CDs possess characteristics such as facile synthesis, small size, good dispersibility, abundant surface functional groups, and ease of functionalization, enabling their applications in biomedicine, light-emitting diode (LED) optics, lubrication, catalysis, agriculture, and other fields [22,84,85,86]. In the past decade, CDs have also demonstrated promising application potential in the field of anti-corrosive coatings. On one hand, CDs can serve as 0D functional fillers that enter micropores, defects, and interfacial regions within the coating, thereby enhancing film densification and prolonging the diffusion path of corrosive media. On the other hand, the active surface groups of CDs are conducive to enhancing interfacial interactions with the resin matrix, improving coating stability, and providing a foundation for constructing high-performance anti-corrosion systems that combine barrier protection with functional responsiveness.

### 5.1. Application Performance of CD-Based Coatings

The application of carbon dots in anti-corrosive coatings exhibits a relatively clear stage-wise evolution. Early studies primarily regarded CDs as 0D functional fillers for enhancing the self-healing capabilities and basic anti-corrosion properties of polymer systems. Subsequently, research focus gradually shifted towards surface functionalization and compounding with 2D materials to further enhance interfacial compatibility, long-term barrier performance, and local active protection effects. In recent years, with deepening functional design, CDs have been endowed with additional features such as fluorescence warning, self-reporting, photothermal response, self-healing, lubrication regulation, and even electrochemical interface stabilization, driving anti-corrosion systems towards intelligence and multifunctionality. Overall, the role of CDs in anti-corrosive coatings is evolving from early-stage reinforcing fillers into key components that integrate structural construction, interfacial modulation, and functional responsiveness.

Research on the application of CDs in anti-corrosive coatings began in 2017. Kang’s team [87] first introduced surface-functionalized CDs into polymer matrices and found that CDs not only enhanced the corrosion resistance of polymer coatings but also endowed the materials with significant self-healing ability. This study incorporated CDs with different surface functional groups into systems such as polymethacrylate (PMMA) and polyurethane (PU). The results showed that under optimal addition, the tensile strength of the healed CD-5/PMMA composite could recover approximately 60% of that of the original sample. In the PU coating, a scratch almost completely disappeared after 48 h at room temperature, while a similar scratch healed noticeably in just 10 min at 60 °C. Following this foundational work, research began to focus on the direct application and functional regulation of CDs in epoxy and waterborne epoxy anti-corrosion systems. Pourhashem et al. [88] functionalized GQDs with a silane coupling agent to obtain f-GQDs and incorporated them into solvent-based epoxy. Electrochemical results indicated that f-GQDs significantly enhanced the corrosion protection performance of the epoxy coating. Subsequently, Ren et al. [89] prepared nitrogen-doped CDs (NCDs) and introduced them into a waterborne epoxy system. The results showed that the coating containing 2.0% NCDs exhibited an impedance modulus 364 times higher than that of the control group after 800 h of immersion in 3.5 wt.% NaCl solution. Later, Pu et al. [19] further demonstrated that an appropriate amount of NCDs facilitated the formation of a denser interfacial layer and passivation layer, thereby enhancing the long-term immersion stability of the waterborne epoxy. The common feature of this stage was that CDs were mainly added to the resin as 0D functional fillers, improving the fundamental anti-corrosion performance by enhancing film densification, inhibiting pore expansion, and strengthening interfacial bonding.

As research progressed, CD-modified anti-corrosive coatings quickly evolved from single resin reinforcement to composite anti-corrosion systems constructed in synergy with 2D materials. Early representative work focused on the combination of CDs with graphene-based sheets. Ye’s team [90] constructed a CD-G/epoxy (EP) composite system via π-π interactions. The results indicated that the introduction of CDs significantly improved the dispersion and interfacial compatibility of graphene in epoxy. Electrochemical and transport property tests showed that the coating containing 0.5 wt.% CD-G exhibited optimal protection, with an oxygen permeability coefficient and water absorption of only 4.27 × 10^−13^ cm^3^·cm·cm^−2^·s^−1^·Pa^−1^ and 4.4%, respectively, after 50 days of immersion. Building on this, Zhao et al. [14] further introduced CQDs into a g-C_3_N_4_/epoxy system. They found that the resulting CQDs@g-C_3_N_4_ composite not only possessed better interfacial compatibility and barrier capability but also endowed the system with a certain degree of active protection via the adsorption of CDs on the metal surface and enabled early microcrack identification through fluorescence response. Guo’s team [91] employed amino-functionalized CDs (FCDs) to intercalate boron nitride nanosheets (BNNS), constructing an FCDs/BNNS waterborne epoxy composite system. The results showed that the impedance of this coating remained significantly higher than that of pure waterborne epoxy after 40 days of immersion in 3.5 wt.% NaCl, indicating that CDs not only facilitated BNNS exfoliation and dispersion but also synergistically enhanced the barrier effect and local corrosion inhibition capability of the layered sheets. These studies demonstrate that in 2D sheet composite systems, the role of CDs is no longer limited to “additional 0D nanoparticles” but has gradually evolved into a key component that integrates dispersion regulation, interfacial bridging, and synergistic protection.

In recent years, the synergistic modification of CDs with 2D layered nanomaterials has become a research hotspot in anti-corrosive coatings, particularly the regulation of dispersion and interfacial effects on sheet materials such as layered double hydroxides (LDH), α-zirconium phosphate (α-ZrP), and MXene by CDs, significantly advancing the development of high-performance composite anti-corrosion systems. Zhao’s team [92] introduced carboxyl-rich CDs into the interlayers of LDH to prepare LDH-Cdot nanohybrids. The results showed that CDs significantly improved the dispersion of LDH in water, with the zeta potential shifting from +6.33 mV to −27 mV and the interlayer spacing increasing from 0.764 nm to 0.892 nm. After incorporating LDH-Cdot into waterborne epoxy, the resulting coating maintained a low-frequency impedance modulus (|Z|_f_ = 0.01 Hz) of 4.05 × 10^8^ Ω·cm^2^ after 90 days of immersion. Similarly, Dai’s team [93] intercalated CDs (LCDs) into α-ZrP to construct an LCD-ZrP/waterborne epoxy (WE) composite system. The impedance of this coating reached 9.97 × 10^9^ Ω·cm^2^ after 28 days of immersion, compared to only 4.74 × 10^6^ Ω·cm^2^ for pure WE, indicating that CDs not only amplified the labyrinth barrier effect of α-ZrP but also formed a coordination protective layer with the substrate through surface groups such as -SH, -OH, -NH_2_, and -COOH. Subsequently, Zhou’s team [94] anchored N-CDs onto the surface of Ti_3_C_2_T_X_ MXene to construct a CDs@Ti_3_C_2_T_X_/EP composite coating. This system maintained a low-frequency impedance of 1.38 × 10^11^ Ω·cm^2^ after 70 days of immersion in 3.5 wt.% NaCl, while the coefficient of friction (COF) was reduced to 0.234 and the wear rate was only 4.87 × 10^−4^ mm^3^/Nm, demonstrating an integrated advantage of long-term anti-corrosion and lubrication performance. Inspired by the predatory mechanism of the Venus flytrap, Zhang et al. [95] further co-grafted CDs and phenanthroline (Phen) derivatives onto MXene to construct a PCD-MX/waterborne epoxy (WEP) smart anti-corrosion system. This coating maintained a low-frequency impedance modulus of 5.83 × 10^8^ Ω·cm^2^ after 90 days of immersion, which was one to two orders of magnitude higher than that of blank WEP. After being buried in soil for 70 days, its impedance remained at 3.61 × 10^7^ Ω·cm^2^ with no obvious corrosion. Density functional theory (DFT) calculations indicated that both [Fe(Phen)_3_]^3+^ and CD@Fe^3+^ complexes possessed good thermodynamic stability, and the hydrogen bonding between PCD-MX and the resin ensured excellent interfacial compatibility. Thus, the synergistic design of CDs with layered nanomaterials has become an important technical route for constructing high-performance anti-corrosive coatings.

**Figure 7:**
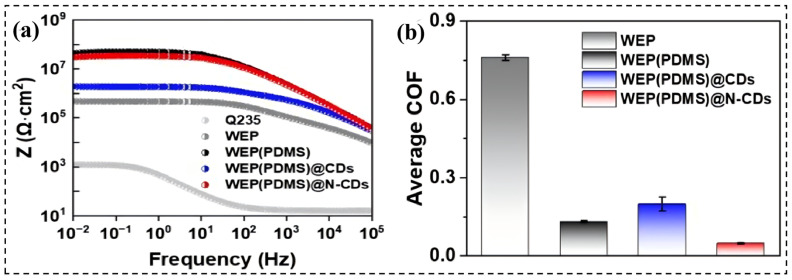
Electrochemical corrosion resistance and dry tribological properties of the as-prepared composite coatings. (**a**) Bode plots of |Z| versus frequency for Q235 steel and WEP-based coatings [96]; (**b**) Average COF of four WEP-based coatings under dry friction condition [96].

Beyond traditional corrosion protection, CDs are progressively being used to impart additional functions to coatings, driving them towards multifunctional smart protection. In terms of friction-corrosion synergy, Qian’s team [96] prepared nitrogen-doped CDs (N-CDs) with a polymer-carbon core hybrid structure and introduced them into a WEP(PDMS) system. The results showed that upon adding N-CDs, the COF of the coating decreased significantly from 0.760 to 0.049, a reduction of 93.6%, while the low-frequency impedance modulus remained at 3.5 × 10^7^ Ω·cm^2^ (Figure 7a,b). In the realm of photothermal response, Wang’s team [97] designed dual-chamber microcapsules with a particle size of approximately 5 μm, using furfurylamine-modified polystyrene-acrylic acid as the shell material and CDs as both the Pickering emulsifier and photothermal-responsive unit. After incorporating these into epoxy, the system could heat up to about 50 °C within 60 s and rapidly close scratches under near-infrared irradiation. The coating containing 2 wt.% microcapsules exhibited high mechanical self-healing efficiency and toughness, and electrochemical tests confirmed its long-term anti-corrosion performance was significantly better than that of the blank epoxy. Another prominent advantage of CDs in anti-corrosive coatings lies in their fluorescence, color response, and interface sensitivity, enabling them to serve as failure monitoring and early warning functions. Compared to fullerenes and nanodiamonds, this feature represents one of the most distinctive differences in CD-based systems. Existing studies have shown that composite systems of CDs functionalized with graphene oxide (GO), g-C_3_N_4_, or layered minerals can exhibit responses such as fluorescence quenching, fluorescence enhancement, or color changes upon coating scratching, metal ion leaching, or under-film corrosion occurrence, thus achieving defect visualization, microcrack identification, and early corrosion warning. For example, luminescent Cs-f-GO/epoxy coatings can visually display scratches and failure areas under UV light [98]; the CD-APhen@SC/PU system can indicate corrosion initiation and propagation through both color change and fluorescence response [99]; and CQDs@g-C_3_N_4_/EP also combines fluorescence monitoring with active/passive protection [14]. Thus, the function of CDs in anti-corrosive coatings has expanded from “improving impedance and delaying corrosion” to a higher level of “damage identification—state feedback—synergistic protection.”

### 5.2. Anti-Corrosion Mechanism of CD-Based Coatings

The mechanism of CDs in anti-corrosive coatings is not simply that of a 0D nanofiller reinforcement but involves multiple aspects, including improved dispersion and enhanced interfacial compatibility, physical barrier and defect sealing, active corrosion inhibition and interfacial passivation, as well as self-healing and smart responsiveness. Compared with traditional inert nanofillers, CDs are typically characterized by small size, abundant surface functional groups, and ease of hybrid modification. Consequently, their role in composite coatings often encompasses both structural construction and functional endowment. Particularly in synergy with 2D materials such as g-C_3_N_4_, BNNS, LDH, α-ZrP, and MXene to construct anti-corrosion systems, CDs not only improve the dispersion state and interfacial bonding of layered fillers within the resin but also provide a certain degree of active protection via surface coordination, adsorption, and complexation. This extends the anti-corrosion mechanism from purely passive barrier to a coupling of passive protection and active response [14,90,91,92,93,94,95].

CDs can significantly improve the dispersion state of fillers in the resin and enhance their interfacial compatibility with the polymer matrix, which is one of the fundamental mechanisms for improving coating protective performance. Early studies have shown that oxygen- and nitrogen-containing functional groups on the surface of CDs can form covalent bonds, hydrogen bonds, and van der Waals interactions with polymer chains, thereby promoting the reconnection of fractured interfaces and enhancing coating integrity [87]. Subsequently, the application of functionalized GQDs, NCDs, and various heteroatom-doped CDs in epoxy and waterborne epoxy further demonstrated that polar groups such as -OH, -COOH, and -NH_2_ on the CD surface are not only beneficial for their own uniform dispersion in the resin but also enhance interfacial interactions with the resin network, increasing crosslinking density and reducing locally loose regions [19,88,89]. For layered fillers such as LDH, α-ZrP, and Ti_3_C_2_T_X_ MXene, the role of CDs is even more prominent. After modification with CDs, the zeta potential of LDH changes from positive to negative, interlayer spacing increases, and aqueous dispersion stability is significantly improved [92]. After intercalating LCDs into α-ZrP, the composite filler exhibits better interfacial bonding with waterborne epoxy, and cross-sectional defects are significantly reduced [93]. In MXene systems, CDs can also enhance the coupling between the sheets and the resin through interfacial connections such as Ti-O-C and C-N, inhibiting agglomeration and interfacial discontinuities [94,95]. Thus, CDs can provide a structural basis for subsequent barrier and active protection through the process of “improving dispersion—enhancing interface—reducing defects.”

CDs can synergize with layered nanomaterials to enhance the physical barrier and defect sealing effects of the coating, which is their most direct anti-corrosion enhancement pathway. CDs typically have dimensions in the nanometer scale, allowing them to enter micropores, microcracks, and locally loose regions formed during resin curing, thereby increasing film densification. When present together with 2D sheets such as g-C_3_N_4_, BNNS, LDH, α-ZrP, and MXene, they can further prolong the diffusion paths of corrosive media like H_2_O, O_2_, and Cl^−^, creating a more pronounced “labyrinth effect” [14,90,91,92,93,94]. On one hand, CDs fill internal micro-defects in the resin; on the other hand, they assist the 2D sheets in constructing longer, more tortuous, and more continuous barrier networks within the coating. Particularly in layered filler systems such as α-ZrP, LDH, and MXene, CDs significantly amplify the inherent physical barrier advantages of the 2D sheets by enhancing sheet dispersion uniformity and interfacial stability. Overall, the role of CDs at this level is not simply “hole filling” but rather systematically improving the coating’s ability to retard corrosive media through the synergistic construction of multi-scale barrier channels with layered nanomaterials.

Beyond passive barrier, CDs can also exert active corrosion inhibition and interfacial passivation functions through adsorption or coordination interactions between surface functional groups and metal ions, which is a key feature distinguishing them from common inert nanofillers. Existing studies have shown that surface functional groups on CDs, such as hydroxyl, carboxyl, amino, thiol, and heteroatoms (N, P, S, etc.), can adsorb or coordinate with metal ions like Fe^2+^/Fe^3+^. This facilitates the formation of a protective adsorption layer or promotes the generation of a local passivation layer at the metal/coating interface, thereby inhibiting charge transfer and local corrosion propagation. For example, LCDs in LCD-ZrP contain functional groups such as -SH, -OH, -NH_2_, and -COOH, which can form an adsorption protective layer on the steel surface, exhibiting a significant corrosion inhibition effect in solution [93]. In the LDH-Cdot system, CDs anchored on LDH can coordinate with metal ions and promote passivation film formation, thereby slowing local corrosion kinetics [92]. In the PCD-MX/WEP system, Phen groups can rapidly form [Fe(Phen)_3_]^2+^ with Fe^2+^, while CDs can also form CD@Fe^3+^ complexes with Fe^3+^, collectively constructing a protective layered structure in defect areas [95]. In systems like CQDs@g-C_3_N_4_/EP, the adsorption of CDs on the metal surface and the resulting active protection trend can also be observed [14]. Thus, a significant advantage of CDs in anti-corrosion systems is their ability not only to delay the ingress of corrosive media to the interface but also to participate in forming a protective layer at the interface, exhibiting a synergistic protection characteristic of barrier and inhibition/passivation.

CDs can also endow coatings with certain self-healing and smart responsiveness capabilities, moving the anti-corrosion system from a static barrier towards dynamic protection. Early studies indicated that interfacial bonding between CDs and polymer chains could promote the reconnection of damaged regions, endowing PU systems with significant self-healing ability [87]. As research deepened, this “healing” mechanism evolved from simple polymer chain reconfiguration to more complex forms, including metal ion capture, complex formation, local protective film reconstruction, and signal response [14,95,97]. Furthermore, the fluorescence, color response, and interface sensitivity of CDs enable them to undertake failure monitoring and early warning functions. Existing studies show that composite systems of CDs functionalized with g-C_3_N_4_ or LDH can exhibit responses such as fluorescence quenching, fluorescence enhancement, or color changes upon coating scratching, metal ion leaching, or under-film corrosion, thus achieving defect visualization, microcrack identification, and early corrosion warning [14,98,99]. It should be noted that the “self-healing” reported in the current literature mostly refers to localized, limited, or auxiliary repair, essentially reflecting inhibition of corrosion propagation rather than complete large-scale structural reconstruction. Nonetheless, the potential demonstrated by CDs in this direction remains significantly stronger than that of most traditional anti-corrosion fillers.

In summary, the mechanism of CD-modified anti-corrosive coatings can be summarized as follows: optimizing coating microstructure by improving filler dispersion and interfacial compatibility; constructing long and tortuous diffusion paths through nano-filling synergy with layered materials; promoting local passivation and active inhibition via adsorption or coordination of surface active groups with metal ions; and further endowing the system with limited self-healing/defect-repair capability and early-warning/sensing functions through fluorescence, complexation, and responsive characteristics. Therefore, the role of CDs in anti-corrosion systems has clearly expanded beyond the traditional 0D nanofiller category, evolving into a multifunctional core component that integrates structural regulation, interface strengthening, active protection, and smart sensing.

## 6. Conclusions and Future Perspectives

To provide a more design-oriented perspective, Table 2 summarizes the main benefits, limitations, optimal loading, suitable systems, and key barriers of the representative 0D carbon-based nanomaterials discussed in this review.

### 6.1. Conclusions

This review systematically summarizes the research progress of 0D carbon nanomaterials in the field of anti-corrosive coatings by integrating quantitative bibliometric analysis with qualitative content analysis. The main conclusions are summarized below from two dimensions: quantitative analysis and qualitative analysis.

(1) From the quantitative analysis perspective, compared with the extensive attention received by 2D carbon nanomaterials (such as graphene and its derivatives) in the field of anti-corrosive coatings, research on 0D carbon nanomaterials started later, and the overall number of publications is relatively small, but it has shown a trend of rapid growth in recent years. Bibliometric analysis based on the WoSCC indicates an overall fluctuating upward trend. Keyword co-occurrence analysis reveals that nanoparticles, performance, and coatings are the core hub nodes in the network of this field, while keywords related to CDs and quantum dots have emerged as the most active research branches. The evolution of burst keywords further reveals that research hotspots have undergone a clear evolutionary path, progressing from early thin film construction and particle application (thin films, particles, water), to mid-stage performance enhancement and surface regulation (mechanical property, surface modification, corrosion resistance), and further to recent composite anti-corrosion systems centered on CDs (CQDs, CDs, composite coatings). This quantitative analysis not only confirms the continuously rising research interest in 0D carbon materials in the field of anti-corrosive coatings but also provides a macro-level background and hotspot focus for the subsequent qualitative analysis.

(2) From the qualitative analysis perspective, this review comprehensively summarizes the three types of 0D carbon nanomaterials from the aspects of performance enhancement and mechanistic contributions.

For ND, their performance enhancement is mainly reflected in the wear-corrosion synergy and interfacial densification. The introduction of ND can significantly reduce the corrosion current density of composite coatings, increase polarization resistance, and generally exhibit a characteristic of “optimal performance at low content” in metal matrix coatings, organic coatings, and sol–gel films. At the mechanistic level, the core contribution of ND stems from its dual structure of a “hard core + flexible surface”: the sp^3^ diamond core provides ultra-high hardness and wear resistance, maintaining coating integrity under friction and erosion conditions, and delaying crack initiation and propagation; the abundant active groups on the surface, such as hydroxyl and carboxyl groups, endow ND with excellent functionalizability, enabling it to form covalent bonds with resin or silane networks, enhancing interfacial bonding and filling micro-defects. Furthermore, the characteristic of ND surfaces that do not readily adsorb Cl^−^ helps inhibit pit propagation, while their electrical insulation effectively reduces the risk of galvanic corrosion when combined with conductive fillers. ND can also serve as pH-responsive nanocarriers for corrosion inhibitors and inhibit dendrite growth at the battery anode interface by establishing high-density nucleation sites. Overall, the anti-corrosion mechanism of ND is a comprehensive manifestation of physical barrier, interfacial enhancement, localized corrosion inhibition, and wear-corrosion synergy.

For fullerenes, their performance advantages have been verified in multiple systems: ion beam-modified fullerene films can reduce corrosion current by two orders of magnitude; fullerene/epoxy coatings remain intact after long-term salt spray exposure; functionalized fullerenes in sol–gel coatings significantly reduce surface roughness and increase polarization resistance; multifunctional composite coatings modified with fullerenes can achieve protection efficiency exceeding 99%; in addition, fullerenes as electric field regulating layers at battery anodes can significantly extend cycle life. At the mechanistic level, the uniqueness of fullerenes lies in their hollow cage structure and sp^2^-hybridized carbon atoms, which impart high electron affinity, free radical scavenging ability, and hydrophobicity. Their anti-corrosion mechanism encompasses multiple aspects: as a physical barrier, fullerenes can fill coating pores and can be transformed into a dense amorphous carbon network after ion irradiation; although the spherical morphology is less effective than high-aspect-ratio fillers in creating tortuous paths, it can synergize with 2D materials to construct complex barrier networks; functionalized fullerenes (oxidized or aminated) can form covalent bonds such as Si-O-C with the sol–gel network, achieving defect repair; as corrosion inhibitor carriers, fullerenes can achieve pH-responsive active release; furthermore, the “nanobearing” effect of fullerenes can enhance the mechanical properties of coatings, while their electric field regulation capability extends to electrochemical interface protection. The anti-corrosion mechanism of fullerenes is a synergistic combination of physical barrier, chemical crosslinking, active corrosion inhibition, and electric field regulation.

For CDs, they exhibit the most extensive functional integration capability in anti-corrosive coatings. In terms of performance, CD-modified coatings can achieve self-healing (scratch closure), long-term high impedance (impedance maintained in the range of 10^8^–10^11^ Ω·cm^2^ after immersion for tens to hundreds of days), ultra-low coefficient of friction (reduction up to over 90%), and fluorescence warning, among other multifunctions. Especially when synergizing with 2D materials (such as g-C_3_N_4_, BNNS, LDH, α-ZrP, MXene, etc.), CDs can significantly amplify the barrier effect of layered fillers, enabling composite coatings to exhibit anti-corrosion performance far exceeding that of single components. At the mechanistic level, the core advantage of CDs originates from their ultra-small size (<10 nm) and abundant surface functional groups (-OH, -COOH, -NH_2_, etc.). These characteristics enable them to: improve the dispersion state of fillers in the resin and enhance interfacial compatibility with the polymer matrix through hydrogen bonding or covalent bonds; fill micropores and defects in the coating, synergistically constructing multi-scale, high-tortuosity barrier networks with 2D materials, thereby prolonging the diffusion paths of corrosive media; form protective passivation layers at the metal/coating interface through adsorption or coordination interactions between surface functional groups and metal ions such as Fe^2+^/Fe^3+^, achieving active corrosion inhibition; utilize fluorescence, color response, and interface sensitivity to generate observable signals upon coating scratching or under-film corrosion, enabling early warning; endow the coating with a certain degree of self-healing capability through mechanisms such as polymer chain reconstruction and metal ion complexation. The anti-corrosion mechanism of CDs has evolved from a single “nanofiller reinforcement” to a multi-level synergistic system of “dispersion improvement—barrier construction—active passivation—smart response.”

In summary, the three types of 0D carbon nanomaterials all demonstrate significantly greater potential than traditional inert fillers in anti-corrosive coatings, but their mechanistic focuses differ: ND excels in “hard barrier + wear-corrosion synergy,” achieving defect filling and mechanical reinforcement through its high-hardness core and surface chemistry; fullerenes excel in “structural regulation + electric field modulation,” achieving densification, crosslinking repair, and interfacial electric field homogenization through their cage-like molecular structure and functionalization capability; CDs excel in “interfacial bridging + smart response,” achieving dispersion enhancement, active passivation, and fluorescence warning multifunctional integration through their ultra-small size and abundant functional groups. From a coating-design perspective, however, 0D carbon nanomaterials should not be regarded as universally superior barrier fillers relative to high-aspect-ratio 1D or 2D nanomaterials. Their practical value lies more in multifunctional and synergistic protection, including micro-defect filling, interfacial enhancement, localized corrosion inhibition, active protection, and smart-response functions; in hybrid systems, they can also cooperate with layered fillers to reinforce long-term barrier performance.

### 6.2. Future Perspectives

Although encouraging progress has been made in the field of anti-corrosive coatings with 0D carbon nanomaterials, several challenges must be addressed to achieve the transition from laboratory research to practical industrial applications. Future research directions can be explored around the following aspects.

(1) Systematic Establishment of Structure-Property Relationships

Currently, for the three types of materials, including ND, fullerene, and CDs, there is a lack of systematic research on the correlations between “structural parameters—dispersion state—coating performance—anti-corrosion mechanism.” The effects of different sizes, types and grafting densities of surface functional groups, and doping elements (N, S, P, etc.) on coating densification, interfacial bonding strength, and long-term protective performance remain unclear. Future efforts should combine controlled synthesis with high-throughput characterization to establish a structure-property relationship database for 0D carbon materials, providing a basis for the rational design of high-performance anti-corrosive coatings.

(2) In-Depth Application of Advanced Characterization Techniques and Multi-Scale Simulations

Current mechanistic studies mostly rely on conventional electrochemical tests (EIS, polarization curves) and static morphological observations, lacking a dynamic understanding of nanoscale evolution processes at the interface. Future research should introduce in situ/operando characterization techniques (such as in situ Raman, in situ AFM, Kelvin probe force microscopy, etc.) to monitor the permeation of corrosive media at the coating/metal interface, local pH changes, and early corrosion nucleation processes in real time. Meanwhile, MD and DFT calculations can be employed to reveal the adsorption/diffusion behavior of species such as Cl^−^, H_2_O, and O_2_ on the surfaces of 0D carbon materials, as well as the coordination mechanisms between functional groups and metal ions, providing theoretical support for mechanistic explanations.

(3) Machine Learning-Assisted Material Screening and Performance Prediction

Due to the vast parameter space for the synthesis of 0D carbon materials (size, doping, surface modification, etc.), traditional trial-and-error approaches are inefficient. Although the experimental data accumulated in this field are currently insufficient, existing data can provide a training foundation for preliminary machine learning models. In the future, standardized experimental databases should be established, and machine learning algorithms (such as random forests, support vector machines, and graph neural networks) should be utilized to predict anti-corrosion performance under different structural parameters, accelerating the screening and optimization of high-performance 0D carbon material fillers.

(4) Development of Multifunctional Integrated Smart Coatings

Existing studies have preliminarily demonstrated the smart properties of CDs, such as fluorescence warning, self-healing, and pH-responsive release, but most remain at the proof-of-concept stage. Future research should further integrate multiple response mechanisms (e.g., pH, temperature, mechanical force, light, electricity, etc.) to develop fully closed-loop smart coatings capable of simultaneously achieving “corrosion detection—active inhibition—damage repair.” Furthermore, combining 0D carbon materials with shape memory polymers, self-healing hydrogels, etc., holds promise for achieving larger-scale structural repair.

(5) Expansion of Application Scenarios to Emerging Fields

This review has indicated that ND and C60 have begun to emerge in interface corrosion protection for batteries (Zn anodes, Sn anodes) and electrocatalysis (carbon layer of CoRu catalysts). Future research should further explore the application of 0D carbon materials in the following emerging areas: (a) integrated pressure- and corrosion-resistant coatings for ultra-deep ocean environments; (b) flexible, stretchable anti-corrosion encapsulation layers for flexible electronic devices; (c) multi-functional coatings combining corrosion resistance and antibacterial properties for biomedical implants; and (d) protective coatings for aerospace applications under high-temperature and strong radiation conditions. These scenarios impose higher demands on the mechanical flexibility, thermal stability, biocompatibility, and environmental adaptability of coatings, for which the multifunctionality of 0D carbon materials provides ideal solutions.

(6) Key Issues in Scalable Preparation and Engineering Applications

Currently, the vast majority of studies are based on small-scale laboratory synthesis, leaving issues such as batch-to-batch stability of nanoparticles, repeatability of dispersion processes, and compatibility with existing coating production lines unresolved. Future efforts should focus on developing low-cost, green, and scalable synthesis routes (such as biomass-derived CDs, optimization of purification processes for detonation ND), and systematically evaluate the failure mechanisms of coatings modified with 0D carbon materials under real service conditions, including long-term outdoor exposure, salt spray, dry-wet cycling, and UV aging. Additionally, establishing unified performance evaluation standards (such as dispersion degree, critical addition amount, and long-term impedance thresholds) is crucial for promoting industrialization.

(7) Synergistic Design of Multi-Dimensional Carbon Nanomaterials

This review focuses on 0D carbon materials, but in practical applications, the combination of 0D with 1D and 2D carbon materials often produces a synergistic effect greater than the sum of their parts (“1 + 1 > 2”). Future research should systematically investigate the spatial arrangement, interfacial interactions, and synergistic anti-corrosion mechanisms of 0D/2D (e.g., ND/GO, CDs/MXene), 0D/1D (e.g., C60/CNT), and 0D/0D (e.g., ND/C60) hybrid systems within coatings. By constructing multi-scale and multi-dimensional barrier networks, it is expected to break through the performance limits of single-dimensional materials.

In summary, although significant progress has been made with 0D carbon nanomaterials in the field of anti-corrosive coatings, the journey from deep mechanistic understanding to practical engineering applications remains long. Future research should be grounded in interdisciplinary approaches (materials science, electrochemistry, computational simulation, data science), centered on structure-property relationships, and guided by the goals of intelligence and multifunctionality, to propel 0D carbon-based anti-corrosive coatings towards higher performance, greater stability, and smarter functionality.

## Figures and Tables

**Table 1:** Top 20 Keywords with the Strongest Citation Bursts.

Keywords	Strength	Begin	End	2012–2026
thin films	3.87	2012	2017	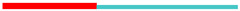
particles	2.79	2013	2018	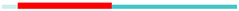
water	4.45	2017	2019	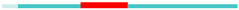
mechanical property	4.01	2018	2022	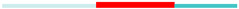
graphene oxide	3.8	2018	2023	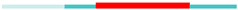
carbon nanotubes	2.94	2018	2022	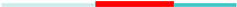
in vivo	2.87	2018	2019	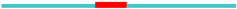
inhibition	2.89	2019	2022	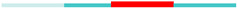
resistance	3.52	2020	2024	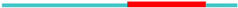
enhancement	3.41	2020	2022	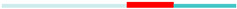
surface modification	3.14	2020	2022	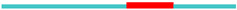
shell	2.98	2020	2022	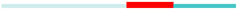
surface	2.71	2020	2021	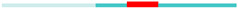
efficiency	3.34	2021	2023	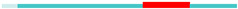
nanocomposites	3.17	2022	2023	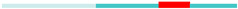
corrosion resistance	3.02	2022	2023	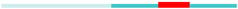
carbon quantum dots	8.34	2024	2026	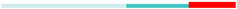
carbon dots	7.39	2024	2026	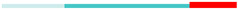
composite coatings	2.89	2024	2026	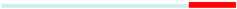
mild steel	2.74	2024	2026	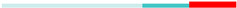

**Table 2:** Design-oriented comparison of representative 0D carbon-based nanomaterials for anti-corrosive coating applications.

Material	Benefit	Limitation	Optimal Loading	Coating System	Industrial Barrier
ND	Defect filling, densification, wear resistance, electrical insulation	Agglomeration at high loading; overloading may reduce protection	Typically ~0.01–1.0 wt.%	Metal matrix, epoxy/WEP, sol–gel, DLC-related films	Dispersion control, scalable functionalization, long-term durability
Fullerenes	Barrier effect, compactness improvement, tribological benefit	Limited barrier effect when used alone; high cost; often needs functionalization	Typically ~0.5–1.0 wt.%	Epoxy, sol–gel, metal matrix, standalone carbon films	Cost, dispersion uniformity, scale-up validation
CDs	Good dispersibility, interfacial enhancement, barrier/smart functions	Structural variability; batch inconsistency; uncertain long-term stability	Typically ~0.5–2.0 wt.%	Epoxy/WEP, 2D hybrid coatings, smart coatings	Reproducibility, standardization, and exposure durability

## Data Availability

No new data were created or analyzed in this study. Data sharing is not applicable to this article.
